# Animal Fat Intake Is Associated with Albuminuria in Patients with Non-Alcoholic Fatty Liver Disease and Metabolic Syndrome

**DOI:** 10.3390/nu13051548

**Published:** 2021-05-04

**Authors:** Manuela Abbate, Catalina M. Mascaró, Sofía Montemayor, María Barbería-Latasa, Miguel Casares, Cristina Gómez, Lucia Ugarriza, Silvia Tejada, Itziar Abete, María Ángeles Zulet, Antoni Sureda, J. Alfredo Martínez, Josep A. Tur

**Affiliations:** 1 Research Group in Community Nutrition and Oxidative Stress, University of the Balearic Islands-IUNICS, 07122 Palma de Mallorca, Spain; manuela.abbate@uib.es (M.A.); c.mascaro@uib.es (C.M.M.); sofia.montemayor@uib.es (S.M.); luciaugarriza@gmail.com (L.U.); silvia.tejada@uib.es (S.T.); antoni.sureda@uib.es (A.S.); 2 Health Research Institute of Balearic Islands (IdISBa), 07120 Palma de Mallorca, Spain; 3 Department of Preventive Medicine and Public Health, University of Navarra, 31008 Pamplona, Spain; mbarberia.3@unav.es; 4 Radiodiagnostics Service, Red Asistencial Juaneda, 07011 Palma de Mallorca, Spain; casaresmiguel@gmail.com; 5 Clinical Analysis Service, University Hospital Son Espases, 07120 Palma de Mallorca, Spain; cristina.gomez@ssib.es; 6 Camp Redó Primary Health Care Centre, 07010 Palma de Mallorca, Spain; 7 CIBER Physiopathology of Obesity and Nutrition (CIBEROBN), Instituto de Salud Carlos III (ISCIII), 28029 Madrid, Spain; iabetego@unav.es (I.A.); mazulet@unav.es (M.Á.Z.); jalfredo.martinez@imdea.org (J.A.M.); 8 Department of Nutrition, Food Sciences and Physiology, Center for Nutrition Research, University of Navarra, IDISNA, 31008 Pamplona, Spain; 9 Cardiometabolic Precision Nutrition Program, IMDEA Food, CEI UAM-CSIC, 28049 Madrid, Spain

**Keywords:** dietary intake, animal fat, NAFLD, metabolic syndrome, albuminuria, albumin-to-creatinine ratio

## Abstract

Non-alcoholic fatty liver disease (NAFLD) and metabolic syndrome (MetS) are associated with chronic kidney disease (CKD). Diet could play a predisposing role in the development of increased albuminuria in patients with NAFLD and MetS; however, published evidence is still limited. The aim of this cross-sectional analysis was to assess whether dietary fats are associated with changes in urinary albumin-to-creatinine ratio (UACR) in 146 patients aged 40–60-years with NAFLD and MetS. Dietary data were collected by food frequency questionnaire; UACR was measured in a single first morning void. Sources and types of dietary fats used in the analysis were total fat, fats from animal and vegetable sources, saturated, monounsaturated, polyunsaturated, and trans fats. One-way analysis of variance was performed to assess differences in dietary fats intakes across stages of UACR. The association between dietary fats and UACR was assessed by Pearson’s correlation coefficient and multivariable linear regression. Patients with increased UACR showed a worse cardiometabolic profile and higher intakes of animal fat, as compared to patients with normal levels of albuminuria. Animal fat intake was associated with mean UACR, independent of potential covariates.

## 1. Introduction

Increased albuminuria, defined as urine albumin-to-creatinine ratio (UACR) ≥ 30–300 mg/g [1], is a well-established early marker of chronic kidney disease (CKD) and a risk factor for cardiovascular disease (CVD) and cardiovascular mortality as well as all-cause mortality in patients with metabolic syndrome (MetS) [2,3,4,5]. Importantly, recent evidence from population-based studies shows that even within the conventional normal range, albuminuria is associated with MetS and cardiovascular risk factors in the general population [3,4,6,7], and that it can independently predict kidney disease progression in individuals with and without diabetes [8].

Non-alcoholic fatty liver disease (NAFLD) is the hepatic manifestation of the MetS, and is also an independent risk factor for CKD, CVD, cardiovascular mortality, and all-cause mortality [9,10,11,12]. Moreover, patients with NAFLD are more likely to have higher levels of albuminuria as compared to patients without [13,14,15]. The manifestation of increased albuminuria in NAFLD can possibly worsen the pathophysiology of CKD and CVD even further, making this population at particular risk.

Unhealthy lifestyle factors may influence the development of albuminuria; however, studies are very limited. In general, poor diet quality, sedentary lifestyle, and obesity have been associated with an increased risk of developing increased albuminuria in participants with normal renal function [16,17,18,19]. On the other hand, weight loss could reduce increased UACR and proteinuria in patients with T2DM and MetS, possibly helped by improved insulin sensitivity, blood pressure, and serum lipid profile [20,21,22].

Studies looking at specific nutrients have mainly focused on proteins and shown that a high intake might contribute to kidney dysfunction in T2DM patients as well as in the general population [23,24,25,26,27]. Increased intakes of proteins from animal sources seem to be more strongly associated with a worse renal outcome as compared with proteins from plants [19,28]; however, such findings have not always been confirmed, with some studies arguing that the total quantity of proteins, rather than their source, is more likely to affect renal health [28,29]. Despite the important role that diet might play in both the prevention and the possible amelioration of albuminuria, evidence is still limited, and more studies are needed.

An important gap in research exists about the role of dietary fats on albuminuria in patients with MetS. In the general population, saturated fats, hence fats from animal sources, have been associated with high levels of albuminuria [30,31]; however, it is unknown whether this association exists in patients with MetS and NAFLD.

The aim of this study is to assess the role of dietary fats intakes on albuminuria in patients with NAFLD and the MetS.

## 2. Materials and Methods

### 2.1. Study Design

The present study is a cross-sectional analysis that uses data from an ongoing multicenter prospective randomized controlled trial on the effects of a customized dietary and physical activity intervention on changes in liver fat deposits over a period of 24 months. The study is carried out by the Research group on Community Nutrition and Oxidative Stress of the University of the Balearic Islands and the Department of Food Science and Physiology of the Faculty of Pharmacy and Nutrition of the University of Navarra, Spain. Baseline data used in the present analysis were collected between October 2017 and November 2019.

### 2.2. Subjects

The present cross-sectional analysis includes 146 patients with a diagnosis of NAFLD by liver ultrasound, aged between 40 and 60 years, with a body mass index (BMI) between 27 and 40 kg/m^2^, and suffering from the MetS, as presenting at least three of the main MetS traits as described in the International Diabetes Federation (IDF) consensus [32]: (1) BMI > 30 kg/m² or increased waist circumference: ≥94 cm in males and ≥80 cm in females; (2) triglycerides (TG) levels ≥ 150 mg/dL (1.7 mmol/L) or specific treatment; (3) reduced HDL cholesterol: <40 mg/dL (1.03 mmol/L) in males and <50 mg/dL (1.29 mmol/L) in females or specific treatment; (4) raised blood pressure (BP): systolic BP ≥ 130 or diastolic BP ≥ 85 mm Hg or treatment of previously diagnosed hypertension; (5) raised fasting plasma glucose (FPG) ≥ 100 mg/dL (5.6 mmol/L) or previously diagnosed type 2 diabetes.

Participants were excluded when they had the following exclusion criteria: documented history of previous cardiovascular disease; documented history of previous liver disease with the exception of NAFLD; concomitant or previous (within 5 years) malignant tumor; previous surgical procedure for weight loss (bariatric surgery); acute febrile illness; concomitant urinary tract infection or post renal hematuria; hemochromatosis; severe/nephrotic-range albuminuria; non-medicated depression and anxiety; chronic abuse of drugs or alcohol; obesity associated with endocrine disease (except medicated hypothyroidism); treatment with steroids; intense physical exercise; pregnancy; unwillingness to provide informed consent.

### 2.3. Ethics

The Ethics Committee of the Balearic Islands (ref. IB 2251/14 PI) and the Ethics Committee of the University of Navarra (ref. 054/2015mod2) approved this trial, which followed the Declaration of Helsinki. All participants were informed of the study and signed a written consent. This study was recorded at ClinicalTrials.gov (number NCT04442620; https://clinicaltrials.gov/ct2/show/NCT04442620; accessed on 14 February 2021).

### 2.4. Anthropometrics and Blood Pressure

Height, body weight, BMI, and waist circumference (WC) were collected by trained dietitians. Height was measured by the nearest millimeter using a mobile stadiometer (Seca 213, SECA Deutschland, Hamburg, Germany) with the patient’s head positioned parallel to the soil (along the horizontal Frankfort plane). Weight was measured using the Tanita MC780P-MA digital segmental body composition analyzer (Tanita, Tokyo, Japan), with the patient wearing light clothes (for which 0.6 kg was subtracted from the total), and bare feet. BMI was calculated by dividing the weight in kg by the square of the height in cm. WC was measured using a measurement tape with the patient standing upright. The measurement was taken in duplicate, and the average of the two measurements was used for analysis. Blood pressure (BP) was measured in the non-dominant arm, with the patient resting in a seated position, using a validated semi-automatic oscillometer (Omron HEM-705CP, Hoofddorp, The Netherlands). The measurement was taken in triplicate, 2 min apart, and the average of the three measurements was used for analysis.

### 2.5. General Data and Medical History

Information on socioeconomics, medical history, use of medication, previous diseases, and smoking habits were obtained from all participants. As for alcohol consumption, participants were asked the average weekly consumption of alcoholic beverages and given the option to answer as either “none”, “<7”, or “≥7”. Those patients who were consuming more than 7 alcoholic beverages a week were excluded if presenting a recorded history of alcohol abuse or if considered to present a drinking problem by their primary healthcare physician. Leisure time physical activity over the previous 12 months was also recorded by means of the validated Spanish version of the Minnesota Leisure Time. Physical activity was expressed as metabolic equivalents of tasks per hour (MET/h) [33,34].

### 2.6. Total Energy and Dietary Fats Intake

Total energy expressed as Kcal per day (Kcal/d), total fat, fats from animal and vegetable sources, saturated fatty acids (SFA), monounsaturated fatty acids (MUFA), polyunsaturated fatty acids (PUFA), and trans fatty acids (TFA), expressed as grams per day (g/d), were derived from the analysis of a validated Food Frequency Questionnaire consisting of 148 food and drink items (148 items-FFQ) [35], which assesses dietary intakes over the previous 12 months.

### 2.7. Blood Collection and Analysis

Venous blood samples from all participants were collected after a 12 h night-time fast in one EDTA sample tube for plasma and one citrate sample tube for serum and centrifugated at 3000 rpm for 10 min. Samples were analyzed for fasting glycemia, glycated hemoglobin (HbA1c), bilirubin, aspartate aminotransferase (AST), alanine aminotransferase (ALT), gamma-glutamyl transferase (GGT), uric acid, urea, creatinine, albumin, total cholesterol, high-density lipoprotein cholesterol (HDL-C), triglycerides (TG), and C-reactive protein (CRP) on the Abbott ARCHITECT c16000 (Abbott Laboratories, Abbott Park, IL, USA), employing specific commercial kits. Low-density lipoprotein cholesterol (HDL-C) was calculated by using the Friedewald Formula [36]. Serum fasting insulin was assayed on the Cobas e411 automated analyzer (Roche, Switzerland), or on the Triturus autoanalyzer (Grifols, Barcelona, Spain), depending on the recruiting center, using either an enzyme-based electrochemiluminescence assay or an enzyme-linked immunosorbent assay kit. A single spot urine specimen collected in the early morning was requested to each participant to measure urinary albumin and creatinine excretion. Urine albumin concentration was determined by immunoturbidimetric assay and urine creatinine by a modified Jaffe method on an Abbott ARCHI-TECT c16000. Urine albumin-to-creatinine ratio (UACR) was expressed as mg/g. Normal albuminuria was defined as UACR < 10 mg/g, mildly increased albuminuria was defined as UACR ≥ 10–29 mg/g, and moderately increased albuminuria was defined as ≥30–300 mg/g.

Insulin resistance index was calculated using the Homeostatic Model Assessment for Insulin Resistance (HOMA-IR) formula by Matthews et al. [37]; estimated GFR (eGFR) was calculated using the Chronic Kidney Disease Epidemiology Collaboration (CKD-EPI) formulas [38] and expressed as mL/min/1.73 m^2^.

### 2.8. Statistical Analyses

Statistical analyses were carried out using the SPSS statistical software package version 25.0 (SPSS Inc., Chicago, IL, USA). Continuous variables were expressed as means ± standard deviation (SD), and categorical variables were expressed as frequencies. Assumption of normality for continuous variables was assessed with the Shapiro–Wilk test and visual inspection of histograms and normal probability plots.

One-way analysis of variance (ANOVA) (equal variance) or Welch’s *t*-test (unequal variance) for continuous variables and χ^2^ test for categorical variables, were used to compare unadjusted means and frequencies of clinical characteristics and dietary intakes of patients stratified by UACR status (normal, mildly, and moderately increased). The Bonferroni test was used for post hoc analyses.

Linear associations between variables were evaluated by Pearson’s correlation coefficient. Covariates with a level of significance (*p*) below 0.05 (two-tailed) were entered in multiple linear regression models to investigate their association with UACR.

Multivariable regression analyses were carried out separately according to type of fat exposure (source of fat: animal and vegetable; type of fat: MUFA, PUFA, SFA, and TFA), adjusted for energy intakes (Kcal/d). Significant models were then further adjusted for gender (male/female), age (years, continuous variable), smoking (yes/no), alcohol consumption over 20 g/d (yes/no), and physical activity (MET/h, continuous variable).

All *p*-values were two-sided, with *p* < 0.05.

## 3. Results

Of the 146 patients with ultrasound proven NAFLD and MetS included in the analysis, 58 were women (39.7%), 24 (16.4%) were current smokers, and 25 (17.1%) were consuming more than 20 g of alcohol a day. Mean ± SD age was 52 ± 7, and mean BMI was 33.71 ± 3.72; 102 (69.9%) patients presented normal levels, 24 (16.4%) mildly increased levels, and 20 (13.7%) increased levels of albuminuria. The prevalence of T2DM was 21.2% (*n* = 31), and of high BP was 34.9% (*n* = 51).

Characteristics of the study cohort as stratified by UACR status are displayed in Table 1. As compared to patients with normal levels of albuminuria, patients with UACR 30–300 mg/g presented a higher diastolic BP, and higher levels of fasting glucose, HbA1c, and HOMA-IR than patients with normal albuminuria. They were also more likely to be male and present higher serum creatine levels than patient with mildly increased albuminuria. Finally, patients with mildly increased albuminuria presented a higher eGFR than those with normal albuminuria. There were no significant differences across UACR status for age, smoking status, alcohol consumption, waist and hip circumferences, body weight, BMI, systolic BP, heart rate, total-cholesterol, HDL-C and LDL-C, TG, prevalence of T2DM, high BP, physical activity levels (expressed as MET/h), and use of medications.

In terms of total energy and dietary fats intakes, as displayed in Table 2, patients with moderately increased albuminuria presented higher intakes of animal fat than those with normal albuminuria. No other significant differences were found across the three groups.

**Figure 1:**
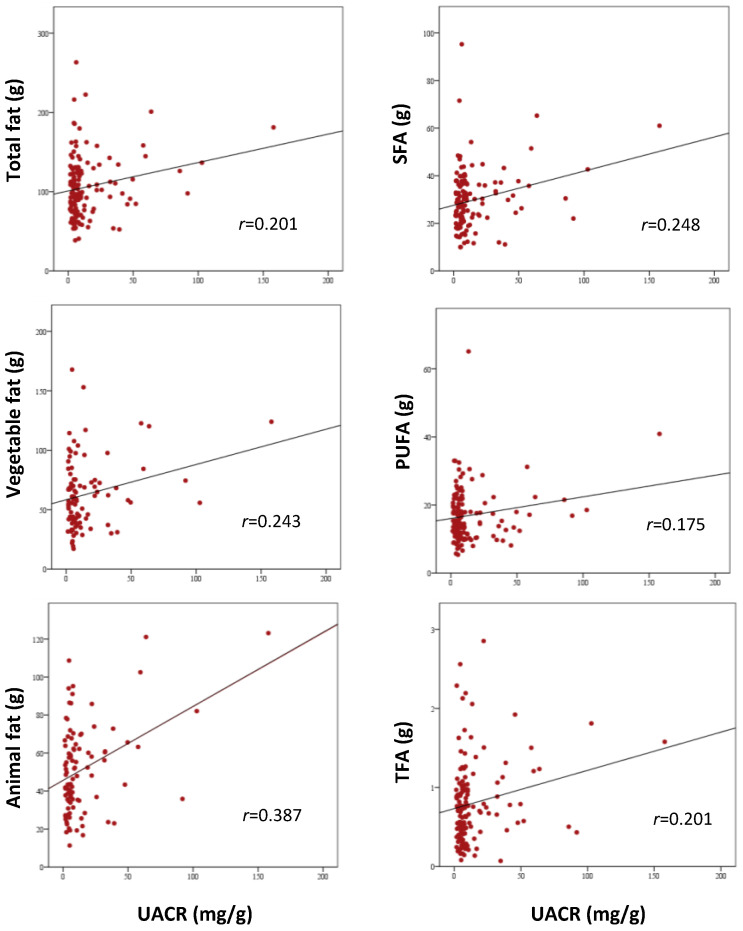
Pearson’s correlation analysis between UACR and dietary fats (all *p* < 0.05). Abbreviations: PUFA: polyunsaturated fatty acids; SFA: saturated fatty acids; TFA: trans fatty acids; UACR: urine albumin-to-creatinine ratio.

As shown in Table 3 and Figure 1, Pearson’s correlation analysis suggested that UACR was significantly correlated with total energy, total fat, fat from animal sources, fat from vegetables sources, PUFA, SFA, and TFA (all *p* > 0.05). Multivariable regression analyses showed that animal fat was the only significant predictor of UACR increase, independently of total caloric intake (*SβC* (standardized β-coefficient) = 0.41, *p* = 0.013; R^2^ = 0.159, *p* = 0.001). When the model was adjusted for gender, age, smoking status, alcohol consumption, physical activity, and treatment with angiotensin converting enzyme inhibitors (ACEi) or angiotensin II receptor blockers (ARBs), animal fat remained significantly associated with increased UACR (*SβC* = 0.43, *p* = 0.013; R^2^ = 0.202, *p* = 0.020).

## 4. Discussion

In the current study, dietary fat from animal sources was significantly associated with increased UACR. The association was independent of mean caloric intake, gender, age, smoking, alcohol consumption, physical activity, and use of ACEi or ARBs. Consistently, consumption of animal fat was higher in patients with moderately increased albuminuria compared to those with normal albuminuria.

In genetic and non-genetic animal models of MetS, consumption of a high-fat diet of mainly animal sources significantly altered the kidney structure and function, inducing increased albuminuria, renal injury, and inflammation, when compared to rats fed a low-fat diet [39,40,41]. Moreover, when quantity of feeding and fat was restricted, further alterations at kidney level were prevented [39]. On the other hand, consumption of unsaturated fats from vegetable sources improved albuminuria, inflammation, and extracellular matrix synthesis in diabetic rats as compared to placebo [42]. At renal level, it was observed that a diet rich in animal fat accelerated renal lipogenesis and suppressed renal lipolysis, with a subsequent accumulation of fat (mainly triglycerides) in both the glomerular and proximal tubules [39,40,43]. In an abnormally metabolic state, excess lipid accumulation in the kidney led to the disruption of the structural integrity of the glomerulus [39,40], induced macrophage infiltration, impaired sodium handling, and increased expression of renin and angiotensinogen [39] and promoted mitochondrial disfunction and, thus, increased oxidative stress and inflammation [43]. In humans, whether lipid accumulation in the kidney is always an etiological factor in the development of glomerular hypertrophy is unknown; however, renal biopsy tissues from patients with T2DM and obesity-related glomerulopathy showed increased lipid deposits in mesangial cells, podocytes, and proximal tubular cells, and most importantly, the extent of the accumulation was proportional to UACR, renal injury, inflammation, and metabolic state [44,45].

Population-based studies observe an association between fat intake, and especially saturated fat, and albuminuria. Specifically, a study on women between the age of 30 and 55 found an odds ratio (OR) of 1.72 (95% confidence interval (CI): 1.12–2.64) when comparing the lowest with the highest quartile of animal fat intake, and an OR of 1.51 (95% CI: 1.01–2.26) when consuming two or more servings/week of red meat for the risk of increased albuminuria [30]. Another cross-sectional study investigating the association between animal fat and early kidney disease in 19,256 participants aged 45 years and older found that saturated fat intake was the only type of fat significantly associated with the presence of high albuminuria (for quintile 5 compared with quintile 1, OR: 1.33; 95% CI: 1.07–1.66) [31]. To the best of our knowledge, no other published data are available on the relation between animal fat intake and albuminuria in the general adult population, and no studies are available in subjects with MetS and NAFLD. Nevertheless, all together, these consistent observations corroborate the hypothesis that dietary fat from animal sources may play a role in the development of albuminuria, and possibly more so when important risk factors such as MetS and NAFLD are already present.

Unfortunately, our data on animal fat could not differentiate between fat from meat and fat from fish. Some studies on protein sources suggest that fish proteins do not affect albuminuria as much as proteins from meat and meat products [28,46], and the same could be true for fat. Several studies on omega-3 polyunsaturated FA supplementation report beneficial effects on urinary albumin excretion and kidney function in patients with and without diabetes [47,48,49,50], possibly by decreasing inflammation and endothelial dysfunction, as well as reducing hypertension and dyslipidemia [51].

Of note, about one third of patients were receiving renin–angiotensin system inhibitor therapy, and it could be speculated that in those patients the detrimental effect of increased animal fat consumption on UACR could be curbed by the renoprotective properties of the therapy [52]. Nevertheless, in multivariate analysis, the relationship between animal fat intake and UACR was independent of ACEi or ABRs therapy.

## 5. Strengths and Limitations

The main limitation of the study is that it was not designed to specifically look at predictors of UACR. Hence, patients were not included according to different stages of UACR, and the vast majority presented levels within the normal range. In fact, only 20 cases presented increased albuminuria. This could explain the weak association, although significant, between UACR and dietary fats. Another limitation is the limited number of patients: a bigger sample could give a more confident answer to the possible relationship between type of fat and the development of increased albuminuria.

## 6. Conclusions

In the current study of patients with MetS and NAFLD, but free of CVD and CKD, consumption of fat from animal sources was associated with increasing levels of albuminuria, independently of mean energy intake and other possible confounding factors. Increased albuminuria is a risk factor for both CKD and CVD, which in turn, weigh heavily on the economic resources of households, health systems, and society [53]. Despite the study’s limitation, these results point out that something as simple as dietary choices could indirectly contribute to the burden.

Patients with MetS and NAFLD are exposed to an increased incidence of a variety of clinical conditions, which could worsen to serious health implications as they manifest and coexist. Besides early screening and appropriate care, it is also fundamental to raise awareness about the effects of dietary choices in the development of chronic diseases. Large intervention studies with prospective dietary measures are needed to elucidate the role of diet in the possible modification of renal vascular dysfunction as an early CVD predictor in patients with metabolic syndrome.

## Figures and Tables

**Table 1:** Differences in patients’ characteristics across stages of UACR.

	UACR < 10 mg/g	UACR ≥ 10–29 mg/g	UACR 30–300 mg/g	*p* *	Post Hoc
*n*	102	24	20		
Age (y)	52.58 ± 8.03	51.17 ± 6.10	52.80 ± 6.07	0.690	
Gender [*n* (%)]				0.010	
Females	41 (40.2)	14 (58.3)	3 (15.0)		b > c
Males	61 (59.8)	10 (41.7)	17 (85.0)		c > b
Alcohol (≥20g/d) [*n* (%)]	20 (19.6)	3 (12.5)	2 (10.0)	0.450	
Currently smoking [*n* (%)]	18 (17.6)	3 (12.5)	3 (15.0)	0.820	
Waist circumference (cm)	111.02 ± 8.74	112.08 ± 9.61	115.79 ± 10.21	0.100	
Weight (kg)	94.21 ± 13.04	94.84 ± 14.62	100.62 ± 15.75	0.160	
BMI (kg/m^2^)	33.27 ± 3.58	34.58 ± 3.35	34.82 ± 4.47	0.110	
Systolic BP (mm Hg)	133.56 ± 13.68	135.90 ± 15.13	141.06 ± 16.97	0.100	
Diastolic BP (mm Hg)	83.88 ± 8.60	86.15 ± 9.78	89.73 ± 10.47	0.030	c > a
Physical activity (MET/h)	20.36 ± 19.48	18.28 ± 19.55	14.06 ± 14.35	0.400	
Fasting glucose (mg/dL)	109.03 ± 25.49	123.83 ± 57.87	148.35 ± 83.04	0.001	c > a
HbA1c (%)	5.93 ± 0.85	6.19 ± 1.46	6.99 ± 2.54	0.005	c > a
HOMA-IR	5.20 ± 3.10	6.80 ± 3.64	9.61 ± 9.53	0.040	c > a
Total cholesterol (mg/dL)	197.73 ± 44.30	196.63 ± 33.24	195.35 ± 36.36	0.970	
HDL cholesterol (mg/dL)	44.74 ± 11.01	43.59 ± 7.14	42.31 ± 10.34	0.600	
LDL cholesterol (mg/dL)	118.72 ± 35.90	114.74 ± 27.16	106.34 ± 31.28	0.340	
Triglycerides (mg/dL)	175.43 ± 125.54	191.50 ± 115.09	239.20 ± 149.50	0.130	
AST (U/L)	25.81 ± 13.28	23.83 ± 9.87	30.06 ± 15.52	0.300	
ALT (U/L)	35.85 ± 30.98	35.67 ± 25.40	44.85 ± 34.22	0.470	
GGT (U/L)	50.37 ± 63.77	49.29 ± 22.29	49.05 ± 30.88	0.990	
Serum creatinine (mg/dL)	0.84 ± 0.15	0.77 ± 0.14	0.92 ± 0.19	0.005	c > b
eGFR (ml/min/1.73m^2^)	85.99 ± 19.28	97.50 ± 14.47	83.78 ± 23.09	0.010	b > a
HBP [*n* (%)]	36 (35.3)	6 (25.0)	9 (45.0)	0.380	
T2DM [*n* (%)]	21 (20.6)	5 (20.8)	5 (25.0)	0.910	
Use of hypoglycemic agents (any) [*n* (%)]	20 (19.6)	4 (16.7)	5 (25.0)	0.783	
Oral hypoglycemic agents alone	19 (18.6)	3 (12.5)	4 (20.0)	0.750	
Insulin and oral hypoglycemic agents	1 (1.0)	1 (4.2)	1 (5.0)	0.372	
Antihypertensive agents (any) [*n* (%)]	36 (35.3)	6 (25.0)	9 (45.0)	0.379	
Diuretic	9 (8.8)	3 (12.5)	4 (20.0)	0.331	
β-Blocker	6 (5.9)	0 (0.0)	2 (10.0)	0.331	
Calcium-channel blockers	5 (4.9)	0 (0.0)	3 (15.0)	0.084	
ACEi/ARBs	31 (30.4)	6 (25.0)	9 (45.0)	0.330	
Lipid-lowering agents (any) [*n* (%)]	29 (28.4)	4 (16.7)	5 (25.0)	0.494	
Statin alone	20 (19.6)	4 (16.7)	3 (15.0)	0.861	
Fibrate alone	7 (6.9)	0 (0.0)	1 (5.0)	0.411	
Statin and fibrate	2 (2.0)	0 (0.0)	1 (5.0)	0.504	

Abbreviations: ACEi: angiotensin converting enzyme inhibitors; ALT: alanine aminotransferase; AST: aspartate aminotransferase; ARBs: angiotensin II receptor blockers; BMI: body mass index; BP: blood pressure; eGFR: estimated glomerular filtration rate; GGT: gamma-glutamyl transferase; HbA1c: glycated hemoglobin; HBP: high blood pressure; HDL cholesterol: high-density lipoprotein cholesterol; HOMA-IR: Homeostatic Model Assessment for Insulin Resistance; LDL cholesterol: low-density lipoprotein cholesterol; METs: metabolic equivalents; T2DM: type 2 diabetes mellitus; UACR: urine albumin-to-creatinine ratio. Data are presented as mean ± standard deviation or counts (%). * *p* obtained by one-way ANOVA (equal variance) or Welch’s *t*-test (unequal variance) for continuous variables and χ^2^ test for categorical variables. Post hoc test by Bonferroni: a = UACR < 10 mg/g group; b = UACR ≥ 10–29 mg/g group; c = UACR ≥ 30–300 mg/g group.

**Table 2:** Difference in fat intakes across stages of UACR.

Mean Daily Intakes	UACR < 10 mg/g	UACR ≥ 10–29 mg/g	UACR ≥ 30–300 mg/g	*p* *	Post Hoc
*n*	102	24	20		
Total Energy (Kcal)	2458.92 ± 840.92	2334.95 ± 533.24	2720.89 ± 978.34	0.300	
Total fat (g)	103.75 ± 37.69	106.88 ± 39.37	116.77 ± 39.03	0.400	
Animal fat (g)	49.35 ± 21.51	47.49 ± 20.30	66.70 ± 31.80	0.030	c > a
Vegetable fat (g)	59.79 ± 27.14	65.36 ± 30.76	72.98 ± 32.67	0.280	
MUFA (g)	49.76 ± 19.68	53.40 ± 19.86	55.55 ± 18.10	0.420	
PUFA (g)	16.68 ± 6.37	17.68 ± 12.10	17.48 ± 7.96	0.820	
SFA (g)	28.82 ± 12.28	28.63 ± 10.63	35.03 ± 14.00	0.120	
TFA (g)	0.75 ± 0.47	0.90 ± 0.64	0.97 ± 0.51	0.130	

Abbreviations: MUFA: monounsaturated fatty acids; PUFA: polyunsaturated fatty acids; SFA: saturated fatty acids; TFA: trans fatty acids; UACR: urine albumin-to-creatinine ratio. Data are presented as mean ± standard deviation. * *p* obtained by one-way ANOVA (equal variance) or Welch’s *t*-test (unequal variance). Post hoc test by Bonferroni: a = UACR < 10 mg/g group; c = UACR ≥ 30–300 mg/g group.

**Table 3:** Correlation and multivariable analyses of the relationship between UACR (mg/g) and energy and dietary fats.

	Correlation Analysis		Multivariable Analysis (1)		Multivariable Analysis (2)	
	*r*	*p*	*SβC*	*p*	*SβC*	*p*
Total energy (Kcal/d)	0.21	0.012	−0.11	0.61	0.03	0.88
Total fat (g/d)	0.20	0.017				
Animal fat (g/d)	0.39	<0.001	0.41	0.013		
Vegetable fat (g/d)	0.24	0.018	0.15	0.31		
MUFA (g/d)	0.12	0.16				
PUFA (g/d)	0.18	0.04			0.04	0.68
SFA (g/d)	0.25	0.003			0.16	0.47
TFA (g/d)	0.20	0.02			0.06	0.65

Abbreviations: MUFA: monounsaturated fatty acids; PUFA: polyunsaturated fatty acids; SFA: saturated fatty acids; TFA: trans fatty acids; *SβC*: standardized β-coefficient. (1): multivariable model including energy, fat from animal sources and fat from vegetable sources as predictors, R^2^ = 0.159, *p* = 0.001; (2): multivariable model including energy, PUFA, SFA, and TFA as predictors, R^2^ = 0.064, *p* = 0.061.

## Data Availability

There are restrictions on the availability of data for this trial, due to the signed consent agreements around data sharing, which only allow access to external researchers for studies following the project purposes. Requestors wishing to access the trial data used in this study can make a request to pep.tur@uib.es.
